# Identification of AAV serotypes for lung gene therapy in human embryonic stem cell-derived lung organoids

**DOI:** 10.1186/s13287-020-01950-x

**Published:** 2020-10-23

**Authors:** Helena Meyer-Berg, Lucia Zhou Yang, María Pilar de Lucas, Alberto Zambrano, Stephen C. Hyde, Deborah R. Gill

**Affiliations:** 1 Gene Medicine Research Group, Nuffield Division of Clinical Laboratory Sciences, Radcliffe Department of Medicine, University of Oxford, Oxford, UK; 2 Department of Biotechnology of Stem Cells and Organoids, Functional Unit for Research into Chronic Diseases, Instituto de Salud Carlos III, Madrid, Spain; 3 Department of Cellular Biology, Functional Unit for Research into Chronic Diseases, Instituto de Salud Carlos III, Madrid, Spain

**Keywords:** human embryonic stem cells, stem cell-based tissue model, gene therapy, rAAV, AAV capsids, AAV serotypes, lung organoids, viral infection model, alveolar type II cells

## Abstract

Gene therapy is being investigated for a range of serious lung diseases, such as cystic fibrosis and emphysema. Recombinant adeno-associated virus (rAAV) is a well-established, safe, viral vector for gene delivery with multiple naturally occurring and artificial serotypes available displaying alternate cell, tissue, and species-specific tropisms. Efficient AAV serotypes for the transduction of the conducting airways have been identified for several species; however, efficient serotypes for human lung parenchyma have not yet been identified. Here, we screened the ability of multiple AAV serotypes to transduce lung bud organoids (LBOs)—a model of human lung parenchyma generated from human embryonic stem cells. Microinjection of LBOs allowed us to model transduction from the luminal surface, similar to dosing via vector inhalation. We identified the naturally occurring rAAV2 and rAAV6 serotypes, along with synthetic rAAV6 variants, as having tropism for the human lung parenchyma. Positive staining of LBOs for surfactant proteins B and C confirmed distal lung identity and suggested the suitability of these vectors for the transduction of alveolar type II cells. Our findings establish LBOs as a new model for pulmonary gene therapy and stress the relevance of LBOs as a viral infection model of the lung parenchyma as relevant in SARS-CoV-2 research*.*

## Background

Recombinant adeno-associated virus (rAAV) is a well-established vector for gene delivery, currently in use clinically for gene therapy, with multiple, naturally occurring serotypes and artificial variants facilitating species-specific cell and tissue tropisms [1]. Engineering of new AAV capsids has been the focus of extensive research, but capsids selected in animal models and cancer cell lines often translate poorly to large animal models and humans. Clinical trials of gene therapy for cystic fibrosis lung disease using AAV serotype 2 failed to show efficacy [2], and of the many potential reasons for this, an important factor is the lack of serotype screening for airway tropism resulting in poor translation to the human airways [3].

The identification of AAV serotypes for gene delivery to the human lung has focused mainly on the transduction of the human airway epithelium [4, 5]. The lung parenchyma, however, is the target for treating genetic diseases such as surfactant deficiencies and interstitial lung disease; in particular, alveolar type II (ATII) pneumocytes, which express proteins crucial for surfactant function.

In this study, we aimed to identify AAV serotypes that permit efficient gene delivery to the human lung parenchyma. We hypothesised that an ideal model for capsid selection should be of human origin and should also offer a polarised cell layer that mimics the tissue surface available to viral vectors, including the distribution of viral entry receptors. We chose a human 3D cell culture model of the lung as a novel approach for serotype screening—lung bud organoids (LBOs) [6]. The LBOs were generated from human embryonic stem cells (hESC) in this study because their differentiation efficiency is more robust compared to iPSC lines [7] as shown specifically for the generation of progenitor lung cells from iPSC [8]. LBOs exhibit a strong bias towards the generation of lung parenchyma cell types, especially alveolar type II (ATII) pneumocytes [6] and provide a reproducible, in vitro model in which to study human/viral vector interactions that is substantially more similar to the native tissue environment than traditional, immortalised, submerged cell culture models. The polarised 3D structure of LBOs allows for vector transduction from the luminal surface, mimicking vector administration by inhalation, and thus provides an attractive translational model for diseases of the human parenchyma.

## Results

**Fig. 1:**
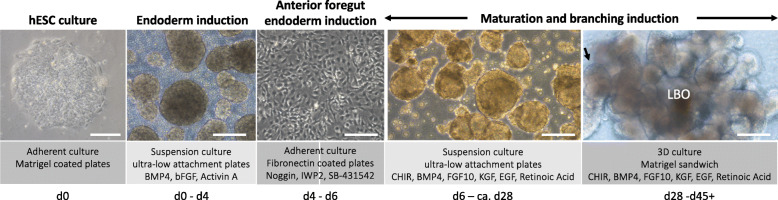
Key stages of lung bud organoid generation. Representative images of the key stages in lung bud organoid generation are shown together with the key respective differences in culture, plate type/coating and cellular factors. d0: hESC are routinely cultured in adherent mode on Matrigel-coated plates. d0–d4: Transition to suspension culture allows differentiation (via embryoid bodies) to definite endoderm. d4–d6: Transition to adherent culture on fibronectin-coated plates and a two-step treatment with cellular factors induces anterior foregut endoderm. d6–d28: Transition to suspension culture allows the generation of nascent organoids. d28–d45+: mature LBOs are generated after nascent organoids are placed in a Matrigel sandwich, with ‘buds’ starting to emerge after a few days as highlighted by the arrow. Mature LBOs are typically used for experiments from d45 onwards (in this study d59 and d79). LBO image shown is representative of d59–79. Images are representative of > 4 cycles of LBO generation. Scale bars 200 μm

**Fig. 2:**
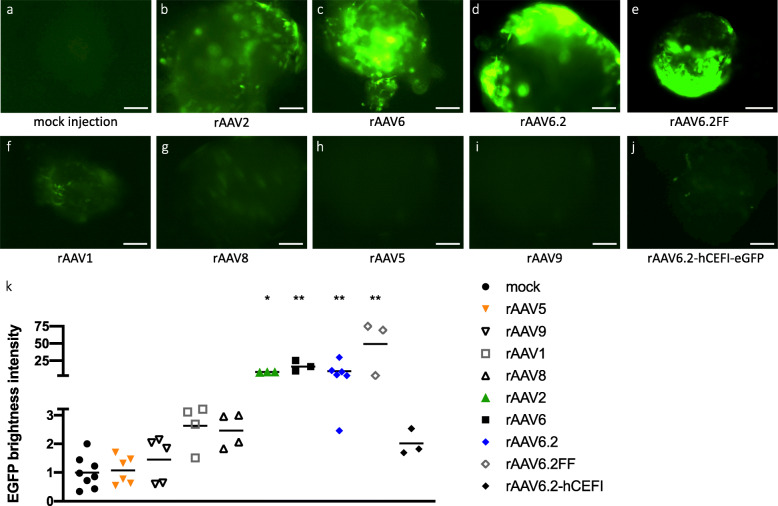
Native EGFP fluorescence in lung bud organoids on day 5 after rAAV transduction. Lung bud organoids (aged d59) were microinjected with 3.5E8 to 1E9 GC of rAAV serotypes expressing EGFP from either the CMV promoter or hCEFI promoter as indicated. Mock injection with buffer served as a negative control. Five days after injection, LBOs were imaged *en face* for EGFP fluorescence (**a**–**j**). One representative image from *n* = 3–4 injected organoids is shown; for serotypes rAAV6.2, rAAV5 and rAAV9, the image is representative of two independent experiments. Scale bar 200 μm. Quantification of EGFP brightness intensity in *en face* images of transduced or mock-injected LBOs (**k**). Brightness was determined as the sum of the values of the pixels in the LBO area of the image and was normalised to LBO size and background fluorescence of the mock injected group. Kruskal-Wallis test comparing the mean rank of each group to the mock injected group. (*) *P* < 0.05; (**) *P* < 0.005

To generate LBOs, the hESC cell line AND-2 was sequentially differentiated via endoderm and branching induction according to the timeline shown in Fig. 1 [6]. After 59 or 79 days of differentiation in culture, LBOs were microinjected with AAV serotypes 1, 2, 5, 6, 6.2, 6.2FF, 8, and 9, or a negative control buffer, to mimic vector delivery to the apical/luminal surface of the lung. Injection of rAAV vectors expressing enhanced green fluorescent protein (EGFP) from the CMV promoter resulted in EGFP-dependent fluorescence in LBOs as early as day 3 post-injection. On day 5 after injection (Fig. 2), high levels of fluorescence directly observed from expressed EGFP (‘native’ fluorescence) were observed following transduction with rAAV2, rAAV6 and variants rAAV6.2 and rAAV6.2FF ([4, 9], Fig. 2b–e, respectively). Native EGFP fluorescence was much lower in cultures injected with rAAV1 and rAAV8 (Fig. 2f, g), while fluorescence with AAV serotypes 5 and 9 (Fig. 2h, i) was indistinguishable from the mock injection (Fig. 2a). For rAAV6.2, EGFP expression from the CMV promoter was considerably more robust than that achieved with the hCEFI promoter [10], which yielded only low levels of fluorescence (Fig. 2d, j). Analysis of EGFP brightness intensity confirmed that expression levels were highest for rAAV2, rAAV6 and variants rAAV6.2 and rAAV6.2FF which was significantly different from mock injection while rAAV5 and rAAV9 resulted in the lowest levels of brightness (Fig. 2k).

**Fig. 3:**
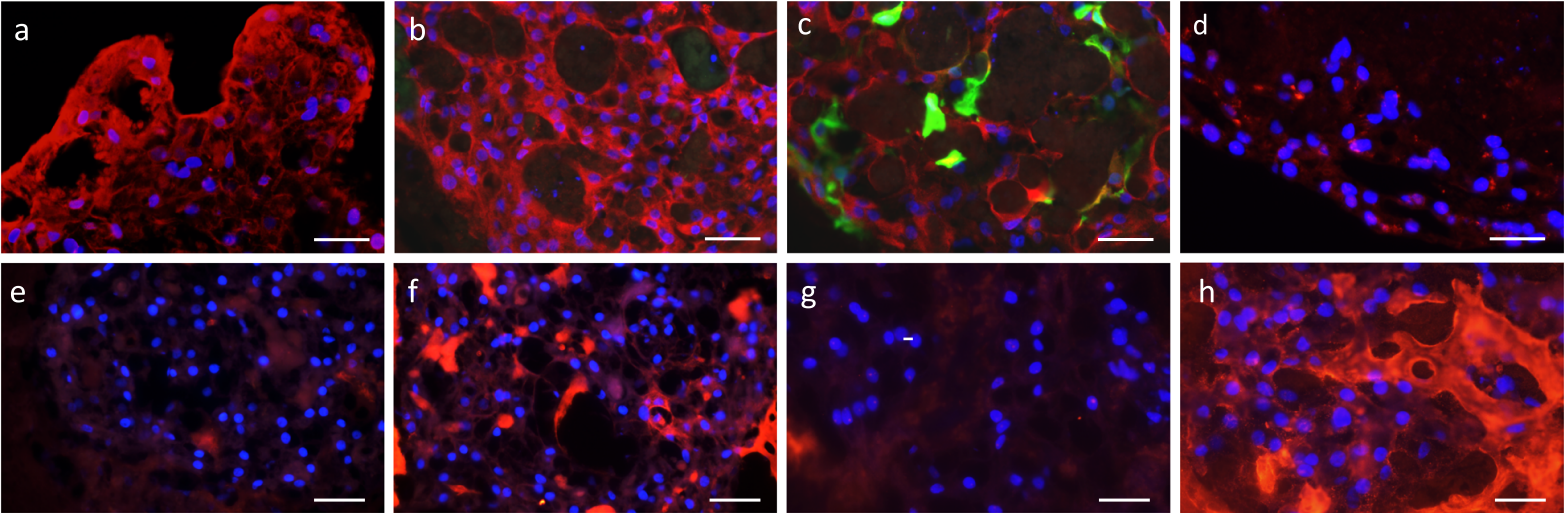
Immunohistochemistry for markers of alveolar type II cells and AAV entry receptors. Representative images are shown of fixed-frozen sections of LBOs (*n* = 2–3), with nuclei stained with DAPI (blue) and various markers (red), including ATII cell marker SP-C (**a**), ATII cell marker SP-B (**b**, **c**), universal AAV co-receptor AAVR (**d**), glycan receptor α-2,3-linked sialic acid (**e**, **f**) and glycan receptor heparan sulphate (**g**, **h**). Native fluorescence is observed following microinjection of AAV6.2-CMV-eGFP (**c**) compared with negative control LBOs (**b**). Sections digested with sialidase A (**e**) and heparinase III (**g**) to remove glycans are included as negative staining controls. Staining controls for images **a**–**d** are shown in suppl. Fig. 2. Scale bar 50 μm

The LBOs were sectioned and stained for ATII cell markers surfactant protein C (SP-C) and surfactant protein B (SP-B) to confirm distal lung maturity (Fig. 3a, b). Native EGFP-dependent fluorescence was observed alongside positive SP-B immunostaining in LBOs transduced with rAAV6.2-CMV-eGFP (Fig. 3c) compared with non-transduced organoids (Fig. 3b) indicating the suitability of this vector to transduce the human lung parenchyma. To further understand the basis for rAAV transduction, which can depend on both primary glycan receptors and protein co-receptors, we also stained the LBOs for the universal AAV co-receptor (AAVR or KIAA0319L*,* Fig. 3d), α-2,3-linked sialic acid (Fig. 3e, f) and heparan sulphate (Fig. 3g, h)*.* Scattered cells staining positive for α-2,3-linked sialic acid were observed (Fig. 3e, f)*,* along with staining of subcellular structures characteristic of AAVR ([11], Fig. 3d) and the ‘spotted’ staining pattern commonly observed for heparan sulphate ([12], Fig. 3g, h). The observations confirmed the presence of all three rAAV receptor molecules in the LBO cultures.

## Discussion

The development of models of the human lung is important for the investigation of new treatments, but is often challenging when human lung tissue is scarce. Moreover, isolated adult alveolar stem cells quickly de-differentiate in culture further complicating their use as founder cells for organoid culture. The generation of LBOs from hESC provides a 3D model of the human lung parenchyma, which has been shown to model aspects of embryonic development, RSV infection and genetic diseases such as Hermansky-Pudlak syndrome [6, 13]. We chose to use this model to identify AAV serotypes with tropism for human lung parenchyma—a crucial step in developing novel viral gene therapies for diseases of the lung.

Microinjection was used to deliver the rAAV vectors to the centre to the LBOs to limit transduction to the apical surface and minimise the opportunity for rAAV transduction via receptors expressed only on the basolateral surface. We showed that reporter EGFP fluorescence was greatest following transduction with AAV serotypes 2, 6 and variants of serotype 6 in d59 organoids, with similar transduction patterns for organoids injected at d79, indicating the robustness of this model over time (suppl. fig. 1). The AAV5 vector was negative for eGFP expression in this human model (Fig. 2h) although serotype 5 was previously identified as suitable for the transduction of murine lung parenchyma [4]. This highlights the variation in vector tropism observed between species, a well-known challenge in the field of viral in gene therapy, and also more generally a problem for viral infection studies. Staining of the LBOs for the lung parenchymal markers SP-B and SP-C confirmed distal lung identity and suggests the suitability of AAV serotypes 2, 6 and variants of 6 for transduction of ATII cells. Serotypes 6.2 (AAV6 capsid + F129L) and 6.2FF (AAV6 + F129L + Y445F + Y731F) were designed for improved lung transduction [4, 9], but in this study, no differences were observed. The hCEFI promoter was investigated for its potential for long-term (months to years) therapeutic transgene expression compared with the strong CMV promoter which is prone to silencing. In a long-term study, native EGFP fluorescence was still detectable with the rAAV6.2 vector on d70 post-transduction for both the CMV and hCEFI promoters (data not shown). Overall, however, hCEFI expression levels were relatively low in the LBOs (Fig. 2k) suggesting that other promoter options for long-term expression in the human lung parenchyma should be explored, such as the Ubiquitin C promoter used in the distal lung [14]. Furthermore, staining revealed the presence of the universal AAVR co-receptor, and also α-2,3-linked sialic acid and heparan sulphate, which have been previously observed in resected adult, human lung tissue [15, 16]. Although the location of these receptors and their subtypes within the human lung and during lung development is not fully understood [12], lectin staining as performed in this study has been reported to intensify in the alveolar region during development from foetal to adult lung [15]. This could lead to improved transduction of adult lung with AAV serotypes dependent on α-2,3-linked sialic acid entry receptors, such as rAAV5, if the glycan receptor rather than a co-receptor was rate-limiting.

The highly efficient transduction of LBOs with AAV serotypes 2 and 6 is consistent with the observed strong positive staining for heparan sulphate (Fig. 3g, h), a key entry receptor for these serotypes [1]. These findings indicate that LBO cultures may be a useful model for screening vectors targeting the human parenchyma, particularly in the early (neonatal) stages of lung development, as required in, for example, treatment of congenital surfactant deficiencies. LBOs might also be suitable for the generation of new capsids targeting the human parenchyma via directed evolution and screening of AAV capsids libraries. The LBOs also have the potential to model aspects of respiratory viral infection in the human parenchyma, including infection of ATII cells with MERS-CoV and SARS-CoV-1 and 2 [17]. LBO sections stained positive for the SARS-CoV-2 entry receptors ACE2 and TMPRSS2 (suppl. Fig. 3), supporting our hypothesis that LBOs may be a useful model for SARS-CoV-2 research.

In future studies, we anticipate that LBOs can be generated from genome-edited hESCs, giving rise to concomitant morphological and functional phenotypes, that could serve as refined human disease models to facilitate the investigation of therapeutic gene therapy vectors. For example, wildtype LBOs have been shown to recycle SP-B from their lumen in a functional assay [6], which could constitute a qualitative read-out of the correction of genetic disorders of surfactant.

In summary, we have established human LBOs as a model to screen for viral vector transduction, identifying serotypes suitable for transduction of the human lung parenchyma.

## Methods

The hESC line AND-2 was sequentially differentiated to LBOs (for details see supplementary methods, Fig. 1 and references [6, 18, 19]). Needles with long continuous taper were pulled for LBO microinjection, which was deemed successful when the organoid visibly pulsated during injection (aiming for 3–4 successful injections per site and, depending on the LBO size and number of buds, 2–3 locations; see supplementary methods for detailed microinjection protocol). This corresponded to 3.5E8-1E9 rAAV genome copies (GC) of vector per LBO (*n* = 3–4 LBOs per group)*.* Recombinant AAV vectors were produced by triple transfection in HEK293T cells and purified via iodixanol density gradient centrifugation [20]. Vector purity was tested via SDS-PAGE and titres were determined using quantitative PCR. For immunohistochemistry analysis, LBOs were processed in fixed-frozen sections. Cellular proteins and glycan receptors were stained using primary antibodies to α-surfactant protein B (#sc-133143, Santa Cruz Biotech), α-prosurfactant protein C (#ab3785, Merck), α-KIAA0319L (AAVR, #PA5-67257, Invitrogen) and α-heparan sulphate, clone F58-10E4 (#370255, Amsbio), as well as *Maackia amurensis* Lectin II to detect α-2,3-linked sialic acid (#B-1265, Vectorlabs). As a negative control for staining, glycan receptors were digested using heparinase III or sialidase A and sections processed in parallel.

## Supplementary information

**Additional file 1: Supplementary figure 1.** Comparison of EGFP brightness intensity in rAAV-dosed d59 vs. d79 organoids. **Supplementary figure 2.** Negative staining controls (with no primary antibody) for immunohistochemistry on fixed-frozen sections in figure 3. **Supplementary figure 3.** Immunohistochemistry in LBO sections for entry receptors of SARS-CoV-2.

## Data Availability

Please contact the corresponding author to request datasets used and analysed in this study are available from the corresponding author on reasonable request.

## Abbreviations

AAV: Adeno-associated virus
AAVR: AAV receptor
ATII cell: Alveolar type II cell
BMP: Bone morphogenetic protein
CMV: Cytomegalovirus
EGFP: Enhanced green fluorescent protein
FGF: Fibroblast growth factor
GC: Genome copies
hCEFI: Human CMV enhancer coupled to EF1α promoter
hESC: Human embryonic stem cell
KGF: Keratinocyte growth factor
LBO: Lung bud organoid
rAAV: Recombinant AAV
RSV: Respiratory syncytial virus
SARS-CoV: Severe acute respiratory syndrome coronavirus
SP: Surfactant protein
