# Supplementary material for: Identification of AAV serotypes for lung gene therapy in human embryonic stem cell-derived lung organoids
Source: Stem Cell Res Ther. 2020 Oct 23;11:448. doi: 10.1186/s13287-020-01950-x (PMC7582027; doi:10.1186/s13287-020-01950-x)
Supplement: Supplementary file 1 — Additional file 1: Supplementary figure 1. Comparison of EGFP brightness intensity in rAAV-dosed d59 vs. d79 organoids. Supplementary figure 2. Negative staining controls (with no primary antibody) for immunohistochemistry on fixed-frozen sections in figure 3. Supplementary figure 3. Immunohistochemistry in LBO sections for entry receptors of SARS-CoV-2. [file 13287_2020_1950_MOESM1_ESM.pdf]

SUPPLEMENTARY

I. METHOD DETAILS

Human Embryonic Stem Cell culture

The hESC line AND-2 was obtained from the "Biobanco de células madre de Granada" (ISCIII, Spain) at passages 29–35 and was karyotyped and confirmed free of mycoplasma contamination. Maintenance and expansion of hESCs were carried out as previously described [1, 2]. Approval for the use of these cells and the experimental procedures were obtained from the ISCIII Ethics Committee and the National Committee of Guarantees for the Use and Donation of Human Cells and Tissues (ref. no. 436 351 1). Mouse embryonic fibroblasts (MEFs) were obtained at 13.5 days post-coitum from C57BL/6J mice as described previously [3].

Sequential differentiation and formation of lung bud organoids (LBOs)

Sequential differentiation of AND-2 cells was carried out as indicated in figure 1 and previously described [1, 2, 4]. MEFs were depleted by passaging AND-2 cells onto MW6 plates (cat. no. 140675, Thermo Scientific) coated with Matrigel (cat. no. 354230, Life Technologies) before sequential differentiation. The first step was the endoderm induction step (days 0-4) which began with formation of embryoid bodies followed by primitive streak formation. The endoderm induction step was performed in serum-free differentiation (SFD) medium composed of a 3:1 mix of IMDM:F12 media (cat. nos. B12-722F and 10–080 CVR, Corning), supplemented with N-2 (cat. no. 17502–048, Gibco; Life Technologies), B27 (cat. no. 17504–044, Gibco; Life Technologies), 2 mM Glutamax (cat. no. 35050–061 Gibco; Life Technologies), 1% penicillin-streptomycin (DE17-602E, Lonza), and 0.05% bovine serum albumin (BSA; cat. no. A7906, Sigma-Aldrich). Media was filtered using a 0.22 µm polyethersulfone membrane (cat. no. 431097, Corning); 50 µg/mL ascorbic acid (cat. no. A4554, Sigma-Aldrich) and 0.04 µL/mL monothioglycerol (stock >97%, cat. no. M6145, Sigma-Aldrich) were

added before use. AND-2 cultures were dissociated by trypsin digest in clumps of 3-10 cells and transferred to ultra-low attachment 6-well plates (cat. no. 3471, Corning) to form embryoid bodies. The culture was maintained in SFD medium supplemented with 10 µM Y-27632, and 3 ng/mL human BMP4 (cat. no. 314-BP, R&D Systems) in a 5% CO2/5% O2/95% N2 environment (Galaxy 48R incubator; New Brunswick) for 24 h. Embryoid bodies were collected, resuspended carefully in SFD medium containing 10 µM Y-27632, 0.5 ng/mL human BMP4 (cat. no. 314-BP, R&D Systems), 2.5 ng/mL human bFGF (cat. no. 233-FB R&D Systems) and 100 ng/mL human activin A (cat. no. 338-AC, R&D Systems) for definite endoderm induction. Cells were fed after 48 h by removing 50% of the medium and replacing this volume with fresh medium.

Anterior foregut endoderm (AFE; days 5-6) was induced as previously described [1, 2]. Embryoid bodies were dissociated into single cells with trypsin. Dissociated cells were transferred to a conical tube containing stop medium (IMDM medium (cat. no. BE12-722F, Corning) supplemented with 50% fetal bovine serum (FBS; F7524, Sigma-Aldrich), 2 mM Glutamax, 1% penicillin-streptomycin) to neutralise the trypsin. Cells were centrifuged for 5 min at 140g, washed carefully twice with SFD medium and counted; 25,000–30,000 cells/cm^2 were plated on fibronectin-coated (F0895, Sigma-Aldrich) 12-well tissue culture plates in AFE induction medium 1 (SFD medium supplemented with 10 µM SB-431542 (cat. no. 1614, Tocris) and 100 ng/mL of NOGGIN (cat. no. 6057, R&D Systems)). After 24 h of incubation, the medium was aspirated and AFE induction medium 2 (SFD medium supplemented with 1 µM IWP2 (cat. no. 3533, Tocris) and 10 µM of SB-431542) was added to the cultures. This process was carried out under normoxic conditions. After AFE formation, cells were briefly trypsinized into small (3–10 cell) clumps and the reaction was halted with stop medium. Cells were then centrifuged for 5 min at 140g and washed carefully twice with an excess of SFD medium.

To induce branching and maturation, the clumps were plated onto ultra-low attachment 6-well plates (cat. no. 3471, Corning) in branching medium (SFD medium containing 3 µM CHIR99021, 10 ng/mL

FGF10, 10 ng/mL KGF, 10 ng/mL EGF, 10 ng/mL BMP4, 50 nM all-trans retinoic acid). These three-dimensional clumps (nascent lung bud organoids) were incubated and fed every other day for approximately 20–25 days. After that, these nascent organoids were embedded into a Matrigel sandwich assembled in MW96 plates. First, 50 µL Matrigel was applied to the well and allowed to gel. Then nascent organoids were collected with a wide mouth plastic Pasteur pipette containing 50% Matrigel diluted in branching media, and immediately transferred onto the first layer of Matrigel. After solidification of this intermediate layer containing the nascent organoids, 50 µL Matrigel was added on top. Medium was changed every 2–3 days by removing and adding media on top of the Matrigel. Growing branching structures were easily visualized under the microscope after 1 or 2 weeks. LBOs were used at the indicated times and as early as d45.

Microinjection of LBOs

Suitable needles for microinjection of LBOs were made on the day of use from glass capillaries with filament (cat. no. BF100-50-10, Sutter instrument) using a needle puller (P-97, Sutter instruments) to create a needle with a long, continuous taper. Settings for the value "heat" were determined by running a ramp test. Puller settings were determined with consultation of the Pipette Cookbook 2018 by Sutter instruments, Chapter 2. After pulling, needles were broken with a second needle at ca. 20% of the taper length from the needle tip to achieve a 25 µm diameter needle. Needles were filled with viral vectors diluted to the same concentration using microloader™ tips (cat. no. 5242956003, Eppendorf).

HEAT	PULL	VEL	TIME
Ramp-10 (here 463)	70	70	170

LBOs were picked-up with the surrounding Matrigel from the 96-well culture plate and transferred on a sterile microscope slide. Microinjections utilised the electronic FemtoJet microinjector, the Injectman Ni 2 micromanipulator (both Eppendorf) and Zeiss inverted microscope (Axio Observer 3).

With the micromanipulator the tip of the needle was manoeuvred as close as possible above the LBO and when both the LBO and the needle tip shared the same focal plane, the needle was slowly lowered until it penetrated the LBO. The position of the needle inside the organoid was adjusted until the organoid visibly pulsated during injection, which was recorded as a successful injection, aiming for 3-4 successful injections per location. Depending on a visual assessment of LBO size and number of buds, 2-3 locations were injected. All microinjections aimed to deliver approximately 3.5E8-1E9 genome copies (GC) of rAAV per LBO. For example, small LBOs were injected at fewer locations and with fewer injections to deliver vector doses at the lower end of the 3.5E8-1E9 GC range. Due to the 3D nature of the lung bud organoids it is difficult to estimate precise cell numbers from images to calculate the MOI, but extrapolating from the nuclei observed in the 12 µm sections from the centre of the LBO, and a diameter of 600-800 µm, there are a roughly estimated 30 000 to 70 000 cells per LBO. This corresponds with an estimated MOI in the E4 to E5 range. Vectors were diluted to the same concentration, with the exception of rAAV6 which was low in titre and required the injection volume to be adjusted. After injection, the LBO was transferred with a Pasteur pipette containing media to a 96-well plate with fresh media. Culture was continued with media changes every 2-3 days.

In culture imaging of LBOs

The EGFP expression by the LBOs was observed *en face,* in the culture plate, with an inverted fluorescence microscope (Leica DMIL LED).

LBO fixation and sectioning

The LBOs were fixed in 4% PFA for 15 min and subsequently dehydrated at 4C overnight in 30 % sucrose in PBS. The fixed and dehydrated LBOs were embedded in 7.5% gelatin/15% sucrose. First, LBOs were incubated for 15 min at 37 °C in 7.5% gelatin/15% sucrose and then transferred to cryomolds containing already solidified 7.5% gelatin/15% sucrose. A third layer of 7.5% gelatin/15%

sucrose was added to completely cover the LBOs. The blocks were cut at -20 C into 12 µm sections in a Leica CM3050 cryostat. Sections were mounted to Superfrost slides (VWR).

Immunohistochemistry

Organoid sections were analyzed by immunohistochemistry. Sections were washed twice for 5min, then incubated in blocking buffer for 2 h at room temperature (see Table for details) and primary antibody was applied in blocking buffer overnight at 4C. For all targets asides from SP-B heat antigen retrieval was performed before blocking (10 mM sodium citrate, 0.05% Tween 20, pH 6.0; 96C/15 min). The secondary antibody was incubated for 1 h at RT in wash buffer/1% BSA. Between incubation steps, slides were washed extensively with wash buffer (4 x 5min). Conditions are summarized in the table below. For each marker 2-3 organoids were stained.

**Target**	**Blocking**	**Primary antibody**	**dilution**	**Secondary antibody**
**SP-B**	PBS/5%BSA/0.1%TX100	α-surfactant protein B (#sc-133143, Santa Cruz Biotech)	1:200	Goat anti-rabbit (cat. no. A-31570, Life Technologies), 1:500
**SP-C**	PBS/1%BSA/5% normal goat serum/0.1%TX100	α-prosurfactant protein C (#ab3785, Merck)	1:200	Goat anti-rabbit (cat. no. A-31570, Life Technologies), 1:500
**AAVR**	PBS/1%BSA/5% normal goat serum/0.1%TX100	α-KIAA0319L (AVVR, # PA5-67257, Invitrogen)	1:75	Goat anti-rabbit (cat. no. A-31570, Life Technologies), 1:500
**Heparan sulfate**	PBS/1%BSA/0.1%TX100	α-heparan sulfate, clone F58-10E4 (#370255, Amsbio)	1:100	Goat anti-Mouse IgM, (cat. no. A-21426, Life Technologies), 1:500

**α-2,3-linked sialic acid**	TBS/1%BSA	Maackia Amurensis Lectin II (#B-1265, Vectorlabs)	1:100	Streptavidin, A594 conjugate (cat. no. S11227, Life Technologies), 1:500
**ACE2**	PBS/2%BSA/0.1%TX100	α-ACE2 (#AF933, R&D Systems)	1:500	Donkey anti-goat (cat. no. A-21432, Life Technologies), 1:500
**TMPRSS2**	PBS/2%BSA/0.1%TX100	α-TMPRSS2 (#ab92323, abcam)	1:500	Goat anti-rabbit (cat. no. A-31570, Life Technologies), 1:500

As a negative control for staining of LBOs, glycan receptors were digested using heparinase III (#AMS.HEP-ENZ III, amsbio) or sialidase A (GK80040, Agilent). Stained sections were mounted with ProLong Gold with DAPI (cat. no. P369635, Life Technologies). Stained sections were imaged using EVOS FL Auto 2 and Leica DM4B fluorescence microscopes.

AAV vector production

Recombinant viral vectors were produced as described previously [5], following triple plasmid transfection of adherent HEK293T cells and harvesting by freeze-thaw. Capsids were isolated by iodixanol density centrifugation and concentrated [5]. Vectors were titrated by determining the number of DNase-resistant GC per ml by qPCR. Purified rAAV was treated with DNAse I (Thermo Fisher Scientific) for 20 minutes at 37°C to remove residual DNA. Samples were then boiled for 10 minutes to release viral genomes. Using the TaqMan Fast Universal PCR Master Mix (Thermo Fisher Scientific) samples were titrated against a WPRE standard curve with known concentration. The primers and probe used were as follows: forward: TGGCGTGGTGTGCACTGT; reverse: CCCGGAAAGGAGCTGACA; probe: FAM-TTGCTGACGCAACCCCCACTGG-TAMRA. The qPCR was performed at 95 °C for 120 seconds followed by 40 cycles of 95 °C for 1 second and 60 °C for 20 seconds. The purity of the rAAV isolate

was examined by SDS-PAGE. Vectors were normalised to 1E10 GC, boiled under reducing conditions and loaded on precast SDS-gels (Bolt™ 12 %, BisTris, Thermo Fisher Scientific). Gels were run at 200 V and stained using Coomassie stain to visualise AAV capsid proteins VP1, VP2 and VP3.

Image analysis for EGFP brightness

Images recorded *en face* of LBOs on day 5 post injection were analysed using Fiji ImageJ. The area of the LBO was selected in the image and subsequently the EGFP brightness intensity was quantified as sum of the values of the pixels in the selected area using the the built in 'RawIntDen' function. EGFP brightness intensity was normalised to LBO size as determined by selected area in ImageJ. To allow for comparison between to experiments, brightness was normalised to the mean EGFP brightness intensity of the mock injected group.

II. Supplementary Figures

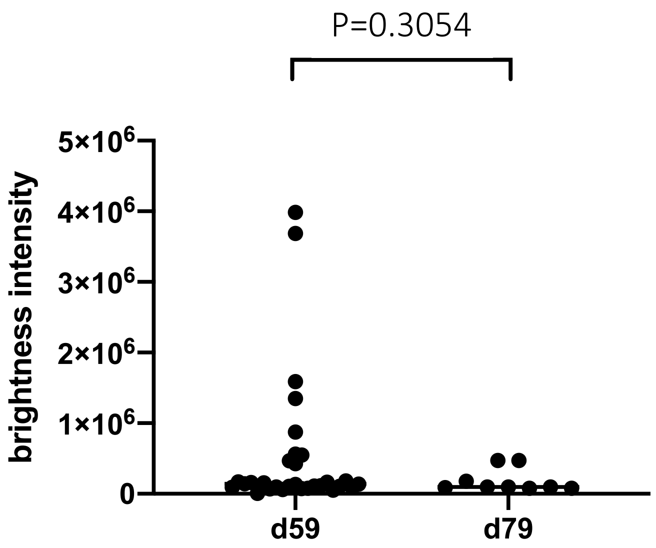

Supplementary figure 1: Comparison of EGFP brightness intensity in rAAV-dosed d59 vs. d79 organoids. EGFP brightness intensity was determined as sum of the values of the pixels in the LBO area of the image and normalised to LBO size. Unpaired t test comparing group means was non-significant.

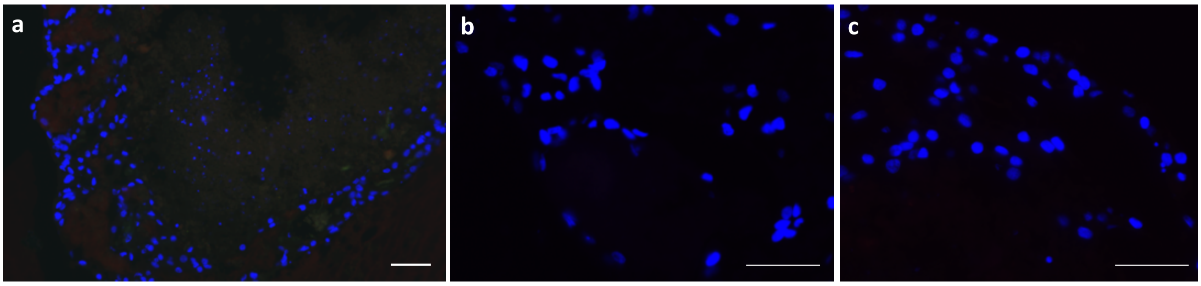

Supplementary figure 2: Negative staining controls (with no primary antibody) for immunohistochemistry on fixed-frozen sections in figure 3.

Negative control for SP-B (a), SP-C (b) and AAVR (c). Scale bars 50 µm.

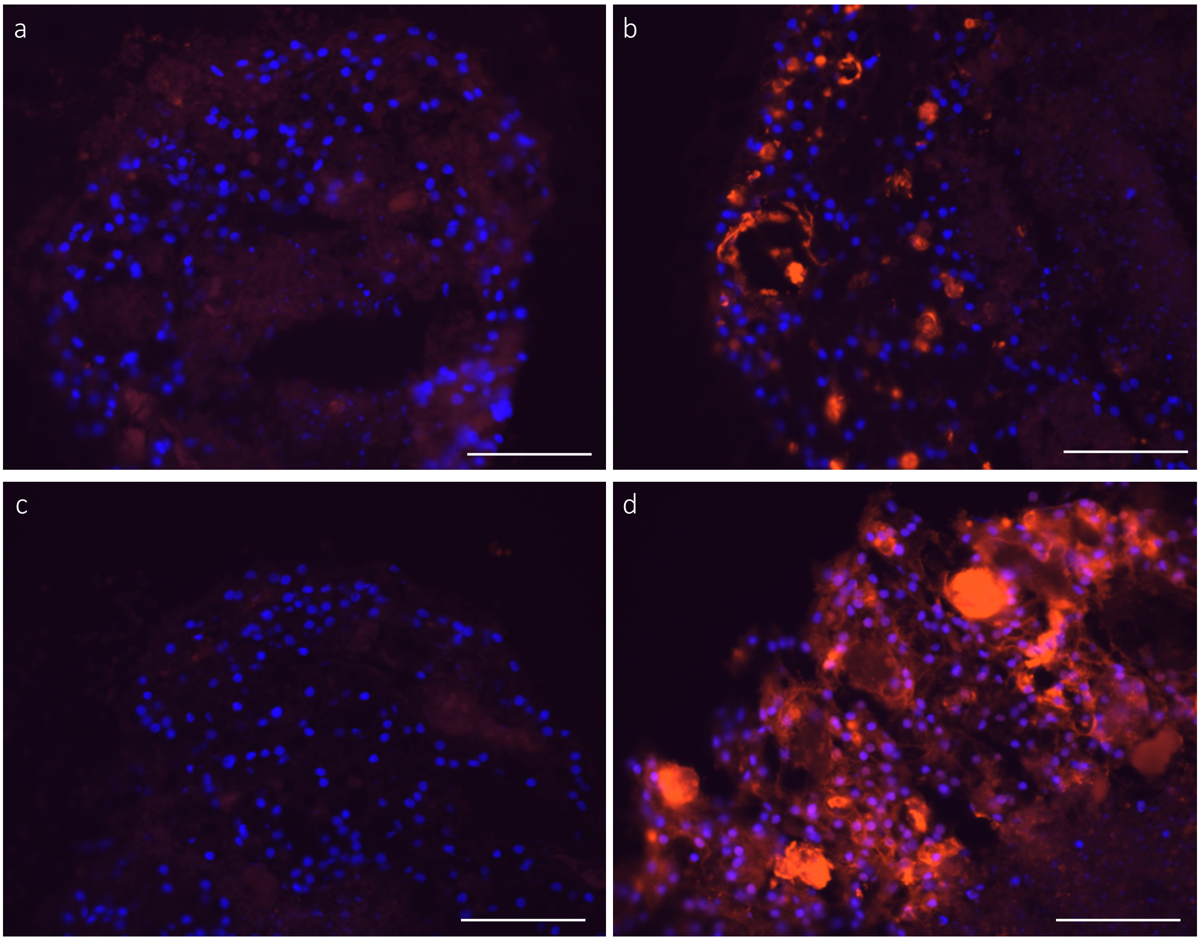

Supplementary figure 3: Immunohistochemistry in LBO sections for entry receptors of SARS-CoV-2. Representative images are shown of fixed-frozen sections of LBOs, with nuclei stained with DAPI (blue) and entry receptors (red), including: ACE2 (b), TMPRSS2 (d). No primary antibody control served as negative staining control for ACE2 and TMPRSS2 (a and c, respectively). Scale bar 100 µm. Representative of n=2 stained organoids.

Bibliograpy

1. Magro-Lopez E, Palmer C, Manso J, Liste I, Zambrano A. Effects of lung and airway epithelial maturation cocktail on the structure of lung bud organoids. Stem Cell Res Ther. 2018;9(1):186.
2. Magro-Lopez E, Guijarro T, Martinez I, Martin-Vicente M, Liste I, Zambrano A. A Two-Dimensional Human Minilung System (Model) for Respiratory Syncytial Virus Infections. Viruses. 2017;9(12).
3. Zambrano A, Garcia-Carpizo V, Gallardo ME, Villamuera R, Gomez-Ferreria MA, Pascual A, et al. The thyroid hormone receptor beta induces DNA damage and premature senescence. J Cell Biol. 2014;204(1):129-46.
4. Chen YW, Huang SX, de Carvalho A, Ho SH, Islam MN, Volpi S, et al. A three-dimensional model of human lung development and disease from pluripotent stem cells. Nat Cell Biol. 2017;19(5):542-9.
5. de Silva SR, McClements ME, Hankins MW, MacLaren RE. Adeno-Associated Viral Gene Therapy for Retinal Disorders. In: Bo X, Verhaagen J, editors. Gene Delivery and Therapy for Neurological Disorders. New York, NY: Springer New York; 2015. p. 203-28.
